# Selective intra-dinucleotide interactions and periodicities of bases separated by K sites: a new vision and tool for phylogeny analyses

**DOI:** 10.1186/s40659-017-0112-0

**Published:** 2017-02-13

**Authors:** Carlos Y. Valenzuela

**Affiliations:** Programa de Genética Humana, Instituto de Ciencias Biomédicas (ICBM), Facultad de Medicina, Universidad de Chile, Independencia 1027, Casilla 70061, Independencia, Chile

**Keywords:** Evolutionary theories, Selective nucleotide interactions, Selective periodicities

## Abstract

Direct tests of the random or non-random distribution of nucleotides on genomes have been devised to test the hypothesis of neutral, nearly-neutral or selective evolution. These tests are based on the direct base distribution and are independent of the functional (coding or non-coding) or structural (repeated or unique sequences) properties of the DNA. The first approach described the longitudinal distribution of bases in tandem repeats under the Bose–Einstein statistics. A huge deviation from randomness was found. A second approach was the study of the base distribution within dinucleotides whose bases were separated by 0, 1, 2… K nucleotides. Again an enormous difference from the random distribution was found with significances out of tables and programs. These test values were periodical and included the 16 dinucleotides. For example a high “positive” (more observed than expected dinucleotides) value, found in dinucleotides whose bases were separated by (3K + 2) sites, was preceded by two smaller “negative” (less observed than expected dinucleotides) values, whose bases were separated by (3K) or (3K + 1) sites. We examined mtDNAs, prokaryote genomes and some eukaryote chromosomes and found that the significant non-random interactions and periodicities were present up to 1000 or more sites of base separation and in human chromosome 21 until separations of more than 10 millions sites. Each nucleotide has its own significant value of its distance to neutrality; this yields 16 hierarchical significances. A three dimensional table with the number of sites of separation between the bases and the 16 significances (the third dimension is the dinucleotide, individual or taxon involved) gives directly an evolutionary state of the analyzed genome that can be used to obtain phylogenies. An example is provided.

## Background

Nearly thirty years ago we undertook the study of the distribution of bases in genomes or chromosomes independently of their location; or structural, functional, coding or non-coding properties. Our aim was to answer the simple question of the neutral (random) or non-neutral (selective) distribution of nucleotides or bases taken at random from genomes, chromosomes or DNA segments, excluding those mentioned properties of genomes. The general context of this aim was to test evolutionary theories from a new global perspective: are nucleotides within genomes neutrally or selectively distributed? We could not find studies with this approach in the scientific literature.

### Searching for tandem repeats of bases

**Fig. 1:**
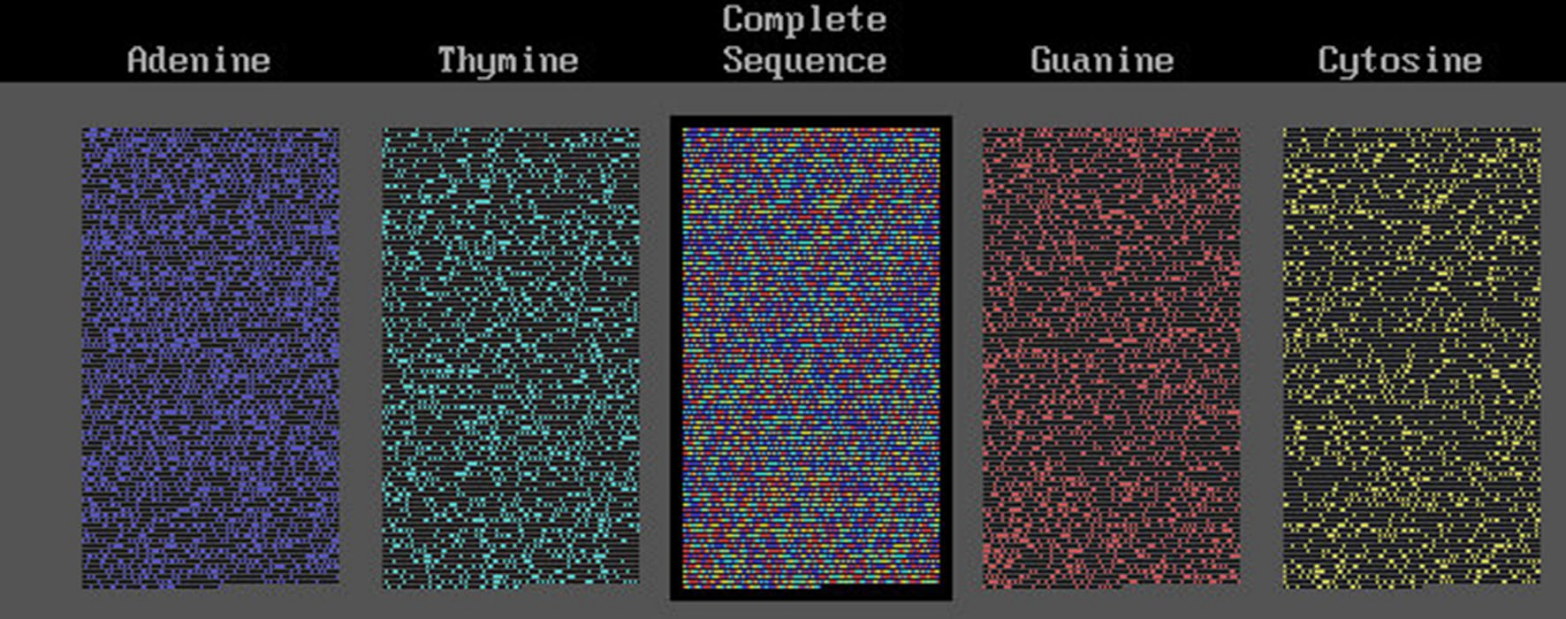
HIV-1 cDNA sequence

The first approach, was related to the proportion of bases and longitudinal distribution of sequences of Adenine (A), Thymine (T), Guanine (G) and Cytosine (C) that are contiguous in sets of 0 (no-base), 1, 2… J bases (tandem series of each base). We needed to solve the problem of the expected random distribution of bases and non-bases in DNA segments. The solution we found for the distribution of nucleotides on chromosomes was the Bose–Einstein (B–E) statistics [1–4]. We applied this statistics to “bases” among “non-bases” and found that they distributed with a B–E statistics in DNA segments, chromosomes or genomes. Bases behaved as indistinguishable balls distributed in distinguishable boxes whose walls were given by the non-base distribution [2, 3, 5, 6]. We applied this distribution to the case of HIV-1 virus and found a huge deviation from the neutral expected distribution of bases in the whole viral “chromosome” [2, 7]. Figure 1 shows the base distribution of this virus: A is in dark blue, T in light blue, G in red and C in yellow; non-bases are in black. The bases of the HIV-1 chromosome distributed far from randomness or neutrality; they appeared to be evolving co-selected or co-adapted as a whole. Some features of the mammal, primate and human genome; such as the deficiency of CpG pairs seemed to be mimicked by the HIV-1 virus. It is remarkable that bases tend to be in sequence clusters; this tendency is more marked in G and C than in A and T which often do not cluster or present the inverse tendency ([2], not published in other species). However, this condition needs more research.

### Searching for non-random internucleotide interactions of bases in dinucleotides

The second approach, which is the present subject, was to study dinucleotides to see whether both bases were neutrally or selectively distributed. To cover all the possibilities of interactions we took all dinucleotides whose bases were separated by 0 (contiguous), 1, 2, 3… K nucleotide sites, in an entire genome or DNA segment [2, 3, 8–13]. If N is the number of nucleotides of a genome, we have N−1 contiguous dinucleotides, N−2 dinucleotides separated by 1 site, N−3 separated by 2 sites… and N−K−1 dinucleotides separated by K sites. The possible dinucleotides are 16, four bases (A, T, G, C) for the first, times four bases for the second nucleotide 0, 1, 2… K sites downstream. We insist on the condition that the location of the first and second nucleotide within any nucleotide sequence, unique or repeated, dispersed or in tandem, functional, coding or non-coding and any other structural properties or base sequences are; not only irrelevant for this study, but they are “intentionally” excluded from the analyses. The only included condition is the number of nucleotide sites between the two bases, but the DNA or RNA sequence between them is completely ignored; it is indirectly included as an average of all the inter-bases sequences of that genome or DNA segment.

## Main text

### Foundation and short description of the method

For any set of dinucleotides, taken from a genome or DNA segment; whose bases are separated by K nucleotide sites, we obtained a summary measure of their difference from neutrality. Here, neutrality is assumed to be the random distribution of the second base in relation to the first base. The rigorous expected random proportion for each base is ¼ (if the four bases have the same selection coefficient the expected proportion for each is ¼ [14–16]), thus 1/16 for each dinucleotide; however, this seems to be an extreme ideal expectancy. Thus; we assumed that the neutral proportion of bases is the observed proportion in the analyzed DNA. This gives the maximal advantage to the neutral hypothesis, because if the observed frequency of the four bases is really selective, this selective condition cannot be ascertained by the method that has included it into its fundamental assumptions (epistemic circularity). Then; the assumed expected dinucleotide proportion is obtained directly as the product of the frequency of the two bases (of the first and the second nucleotide, respectively; they are equal with the exception of the last nucleotides between them which are not included in the analyses). If f1A, f1T, f1G and f1C are the observed frequencies of the bases of the first nucleotide, f2A, f2T, f2G and f2C are the observed frequencies of the bases of the second nucleotide, and D is a generic base, the expected frequency of the dinucleotide is directly f1D × f2D. The expected number of dinucleotides is obtained by multiplying this expected frequency by the number of dinucleotides whose separation between both bases is 0, 1, 2… K sites. The statistical test to evaluate the distance to neutrality is the Chi square test (*χ* ^2^) given by $\sum\nolimits_{1}^{16} {[(O_{i} - E_{i} )^{2} /E_{i} ]} ,$ i between 1 and 16; with 9 degrees of freedom (df) given by 3 df for the first and the second base, respectively; one df is lost in rows and columns because fA + fT + fG + fC = 1. The expected 0.05 or 5% significance level of $\chi_{9}^{2}$ is 16.9 (rounded to 17). We can obtain a $\chi_{1}^{2}$ value for each pair with the respective term of the addition or its particular contribution to the total test. This is an underestimated value because it does not include the value of the complement to the total addition of values; this complement is always much smaller than the so calculated value and may be neglected; in this case the 5% confidence $\chi_{1}^{2}$ value is 3.84. With this method we discovered an enormous deviation from the expected random dinucleotide proportion and periodicity in the value of the total deviation and in the specific deviation of each pair [2–4, 7–13].

The description of the behavior of a particular dinucleotide allows us to understand better the nature of this periodicity. The behavior of the two bases of a dinucleotide is better understood as follows: the random (neutral or nearly) expectancy of the bases predicts that the same or nearly the same number of dinucleotides occur when their bases are separated by (3K), (3K + 1) or (3K + 2) sites (there is no other neutral or nearly neutral expectancy because bases are chosen at random without any reference to functional or structural properties); however, analyzing the *M. smithii* genome ([13], Table 6) we found 1,452,629 CG pairs whose bases are separated by 0–32 sites. The expected number of CG pairs whose bases are separated by (3K), (3K + 1) and (3K + 2) is then 484,209.7, but the observed numbers of pairs are: 413,392 (CG− enormously and negatively selected pairs); 579,517 (CG+ enormously and positively selected pairs); and 459,720 (CG− moderately and negatively selected pairs), respectively. The $\chi_{1}^{2}$ value due to the deviation of the positively selected CG pairs alone is greater than 18,759, this implies a probability P < 10^−1000^, see the following sections. Since in the development of a new field there is possibility of hidden errors, false mathematical models, program errors or other unknown errors, we should consider the history of these programs. First, these programs were elaborated in BASIC nearly 30 years ago, by the author. Twenty years ago an under graduate medical student, knowing the formulae, elaborated almost independently a program in Q-BASIC and a method to obtain figures from the screen. Four years ago another under-graduate student elaborated independently a program written in Java and new software to obtain figures; and finally the author developed a new program written in Python. All the programs have yielded the same results. This history suggests that the results are reliable, although we should wait for studies performed by other scientific groups.

### An example may show the main elements and traits of the analysis

Let us apply our analysis to a hypothetical sequence of one thousand bases with a tandem repeat “ATGC ATGC ATGC ATGC… and so on” until 250 repeats. Let us study only the first 100 dinucleotides or pairs (allowing for the shift of the end nucleotide to complete 100 pairs). With 0 separations (contiguous bases) we have only four pairs AT, TG, GC and CA each one repeated 25 times and the other 12 pairs repeated 0 times. The expected number of pairs is 6.25 for every pair, because there are 100 dinucleotides and each base occurs (randomly or neutrally) with probability 0.25. In Table 1, where we compute the $\chi_{9}^{2}$ test (for 0 site separation), there are twelve boxes with 0 dinucleotides that are negatively selected (−){12 × (6.25 − 0)^2^/6.25 = 75}; and four boxes with 25 dinucleotides that are positively selected (+){4 × (6.25 − 25)^2^/6.25 = 225}; the total is $\chi_{9}^{2} = 75 + 225 = 300$, a highly significant test (P < 10^−15^). Let us compute for 1 site separation; the pairs are now AG, TC, GA and CT repeated 25 times and the other 12 pairs repeated 0 times; then the $\chi_{9}^{2}$ test will be again 300. With 2 sites separation the pairs are: AC, TA, GT and CG, 25 each one and 0 the remaining pairs, the $\chi_{9}^{2}$ test will be again 300; with 3 sites separation the pairs are AA, TT, GG and CC, 25 each and the other pairs are 0 and the test will be 300. The four sites separation is equal to the 0 site separation and the cycle is repeated n (25) times. The $\chi_{1}^{2}$ contributions are given equally by the four positively selected dinucleotides (25 − 6.25)^2^/6.25 = 56.25 and the twelve negatively selected ones (0 − 6.25)^2^/6.25 = 6.25. We see that this periodic base sequence does not show a periodic value of the $\chi_{9}^{2}$ test; on the contrary the series of $\chi_{9}^{2}$ values is invariant: 300, 300, 300, 300… This shows that periodic base sequences have nothing to do with periodicities of the $\chi_{9}^{2}$ value. DNA segments like the one analyzed do exist in hundreds or thousands in any eukaryote genome which is why the global significance of a genome reaches $\chi_{9}^{2}$ values of hundreds of thousands or even millions.

**Table 1:** The $\chi_{9}^{2}$ analysis of 100 dinucleotides of 25 ATGC tandem repeats

2° Base	0 Separation									
	Adenine		Thymine		Guanine		Cytosine		Total	
1° Base	Exp	Obs	Exp	Obs	Exp	Obs	Exp	Obs	Exp	Obs
Adenine	6.25	0	6.25	25	6.25	0	6.25	0	25	25
Thymine	6.25	0	6.25	0	6.25	25	6.25	0	25	25
Guanine	6.25	0	6.25	0	6.25	0	6.25	25	25	25
Cytosine	6.25	25	6.25	0	6.25	0	6.25	0	25	25
Total	25	25	25	25	25	25	25	25	100	100

Let us demonstrate that polymorphic haplotypes or base sequences are not related to our analyses. Imagine that there are two haplotypes in the population; one haplotype is the tandem repeat ATGC ATGC… ×250 and the second haplotype is completely different with the tandem GTAC GTAC… ×250. In the second haplotype, with 0 site separation the dinucleotides are now: GT, TA, AC and CG repeated 25 times (in 100 dinucleotides) and the other 12 dinucleotides are absent; the series with bases separated by 1 site gives GA, TC, AG and CT dinucleotides repeated 25 times and the other 12 dinucleotides are absent, and so on for separations of 2, 3… sites. The analysis for these completely different haplotypes gives the same result 300, 300, 300, 300… This demonstrates that sequence periodicities are not related to this type of stochastic periodicity, with the exception of obvious mathematical relationships (multiple of 3 or other related mathematical functions, as we shall see in the collagen gene). There are 24 (4!) sets of four bases whose tandem repeat yields the same results. Base sequences are not relevant and this test is blind to them. However, this test is extremely valuable to discover systematic relationships of bases beyond their sequences (selective or non-neutral trans-sequence relationships).

### The $\chi_{9}^{2}$ value measures the difference from neutrality and fits the Wright’s adaptive peaks

The $\chi_{9}^{2}$ is a summary value of the deviation from neutrality of this whole genome or given DNA segment for this particular set of dinucleotides whose bases are separated by K sites. This is a measure of how distant from neutrality or how selective (non-neutral) this genome is. Once this measure is obtained it is impossible to search for the specific sequences that are involved in it, because all the nucleotide sequences have been sent to a grinding machine that destroys them conserving only the site number of nucleotides to calculate the distance in nucleotide sites between two of them. We have only one selective value for that genome or DNA segment (the $\chi_{9}^{2}$ value) and one selective value (the $\chi_{1}^{2}$ value) for each of the 16 classes of dinucleotides. These sets of Chi square values typify an adaptive condition or perspective of this genome or DNA segment. This adaptive condition of genomes or DNA segments coincides conceptually with a Wright adaptive peak in the adaptive landscape [3, 4, 17–19]. However, a very important conceptual difference must be remarked; the Wrightian shift of the peaks in this landscape could be due to “random” drift; while in the present analyses the only possible process that can lead to such huge differences from neutrality is a series of selective non-random historical contingencies. These contingent events have been assumed non-critically to occur at random, but, evolutionary contingencies seldom occur, randomly [3].

### The case of *Drosophila melanogaster* mtDNA

Table 2 shows the analysis for the mtDNA of *Drosophila melanogaster* (taken and adapted from [8] and [10], see the figure of base distribution in [10]). We observe the enormous deviation from neutrality of the total set of dinucleotides from 0 to 17 sites of separation. As was mentioned the significance level at 5% for the total $\chi_{9}^{2}$ is 17, and for the individual pair contribution ($\chi_{1}^{2}$) is 3.84. We see $\chi_{9}^{2}$ values from 37 to 485 out of the range of any current Chi square table or program. In these “out of the range values” we estimated the significance knowing that the expected Chi square value is equal to the df and the variance equal to 2df. Thus, we approximated the significance value by using the normalization (Gaussian) of the Chi square distribution according to the number of standard deviations from the mean value. With 9 df, the error included in this approximation is not large, and may be neglected. We approximated the significance by assimilating one decimal point of significance for every 2 standard deviations (a very conservative criterion) equal to $2\sqrt[2]{18} = 8.49$ (we rounded it to 10) over the mean (9). The first value 485 is equivalent to 112.2 standard deviations (SD) from the mean; thus the significance value with probability of occurrence at random is $P = 10^{ - 56.1}$. The minimal value 37 gives *P* < 10^−6^ (from tables or programs). These huge values, of the deviation from neutrality of the distribution of random dinucleotides (they are replaced in this study by all the possible dinucleotide that is the maximum random sample) from the total mtDNA, lead to the conclusion that no neutrality or near-neutrality is possible in this genome, as far as bases of dinucleotides separated by 0, 1,… 17 sites are concerned. Every base is co-adapted with every base of the remaining (residual) genome. Is this deviation restricted to 17 sites of separations? Our study showed that significant $\chi_{9}^{2}$ values were found up to 2000 and more sites of separation [10, 13]; thus these interactions cannot be produced by coding or non-coding functions or any structural restrictions (this DNA has less than 20,000 bp, most of it is coding DNA and both strands are coding strands. Large separations imply that the first base is in one coding segment and the second is in another coding segment). We have studied the behavior of each dinucleotide and all of them show significant interaction and periodicity [13]. The significant interactions and periodicities are not homogeneously distributed along the chromosome; they may vary so as to find DNA segments where dinucleotides are randomly distributed; this heterogeneity has been studied and described [10].

**Table 2:** Total $\chi_{9}^{2}$ values and its $\chi_{1}^{2}$ contribution of the most significant dinucleotide

Sep	$\chi_{9}^{2}$	1° Pair		2° Pair		3° Pair		4° Pair		5° Pair	
		Pair	$\chi_{1}^{2}$Co	Pair	$\chi_{1}^{2}$Co	Pair	$\chi_{1}^{2}$Co	Pair	$\chi_{1}^{2}$Co	Pair	$\chi_{1}^{2}$Co
0	485	(GG)↑	124	(CC)↑	113	(GT)↓	91	(GC)↑	50	(TT)↑	28
1	94	(CG)↑	36	(CC)↑	25	(CT)↓	12	(AG)↓	6	(TC)↓	4
2	405	(GG)↑	116	(CC)↑	106	(GC)↑	33	(CG)↑	25	(TG)↓	23
3	114	(GC)↑	23	(AA)↑	22	(TT)↑	15	(TA)↓	11	(CC)↑	9
4	47	(CG)↑	20	(AG)↓	8	(AT)↑	6	(CT)↓	4	(GT)↓	2
5	381	(GG)↑	139	(CC)↑	51	(AG)↓	32	(GC)↑	32	(CG)↑	30
6	87	(GC)↑	38	(TA)↑	14	(TC)↓	12	(GA)↓	6	(AA)↓	5
7	37	(CG)↑	17	(CT)↓	7	(TG)↓	3	(GG)↑	3	(TT)↑	2
8	375	(GG)↑	149	(CC)↑	45	(CG)↑	36	(GC)↑	29	(TG)↓	24
9	76	(GC)↑	34	(GA)↓	16	(TC)↓	12	(GG)↑	8	(CG)↓	2
10	49	(CG)↑	28	(AG)↓	6	(CT)↓	6	(AT)↑	4	(TA)↑	1
11	367	(GG)↑	144	(GC)↑	45	(CC)↑	35	(CG)↑	26	(AG)↓	23
12	65	(GC)↑	34	(GA)↓	13	(TC)↓	8	(CG)↓	3	(CA)↑	2
13	70	(CG)↑	38	(AG)↓	13	(AA)↑	5	(GC)↓	3	(CA)↓	2
14	310	(GG)↑	78	(CG)↑	48	(GC)↑	44	(CC)↑	32	(GA)↓	22
15	60	(GC)↑	34	(GA)↓	10	(CG)↓	6	(TC)↓	3	(CT)↑	2
16	52	(CG)↑	27	(AG)↓	12	(AA)↑	3	(CT)↓	2	(CA)↓	2
17	322	(GG)↑	91	(CG)↑	45	(CC)↑	40	(GC)↑	22	(AA)↑	22

Separations from 0 to17 sites. *D. melanogaster* mtDNA

Sep, number of separation sites; $\chi_{1}^{2}$Co, $\chi_{1}^{2}$ contribution of this pair to the total $\chi_{9}^{2}$ value (in integers); ↑ more observed than expected pairs; ↓ less observed than expected pairs

### Some particular DNA segments chosen for their known organizational properties

#### Eukaryote DNA segments

**Fig. 2:**
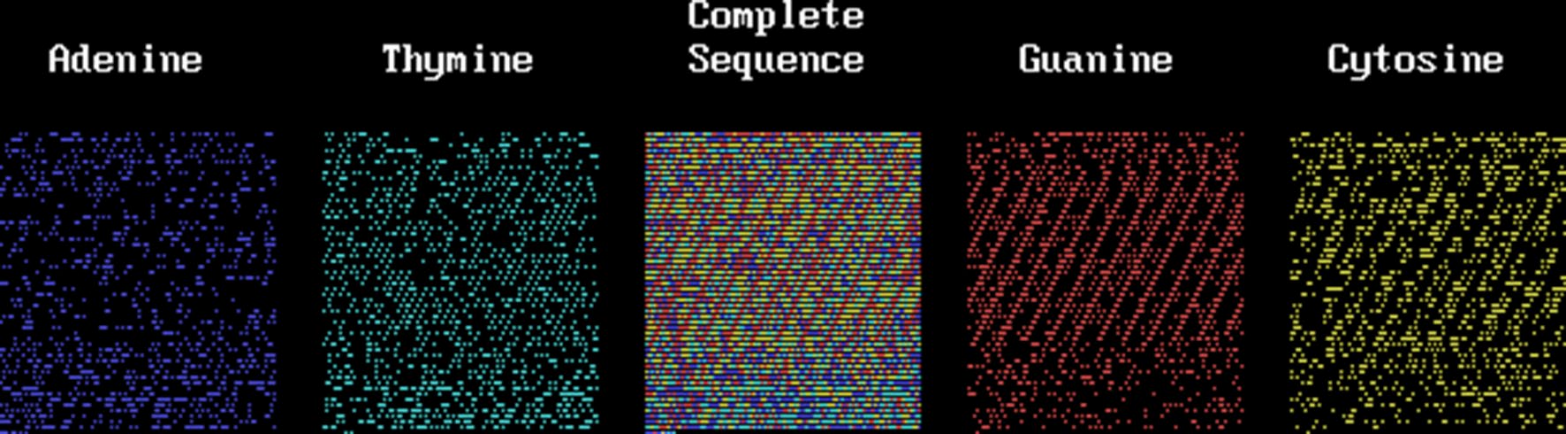
The *Homo sapiens* collagen type I alpha 2 gene

**Fig. 3:**
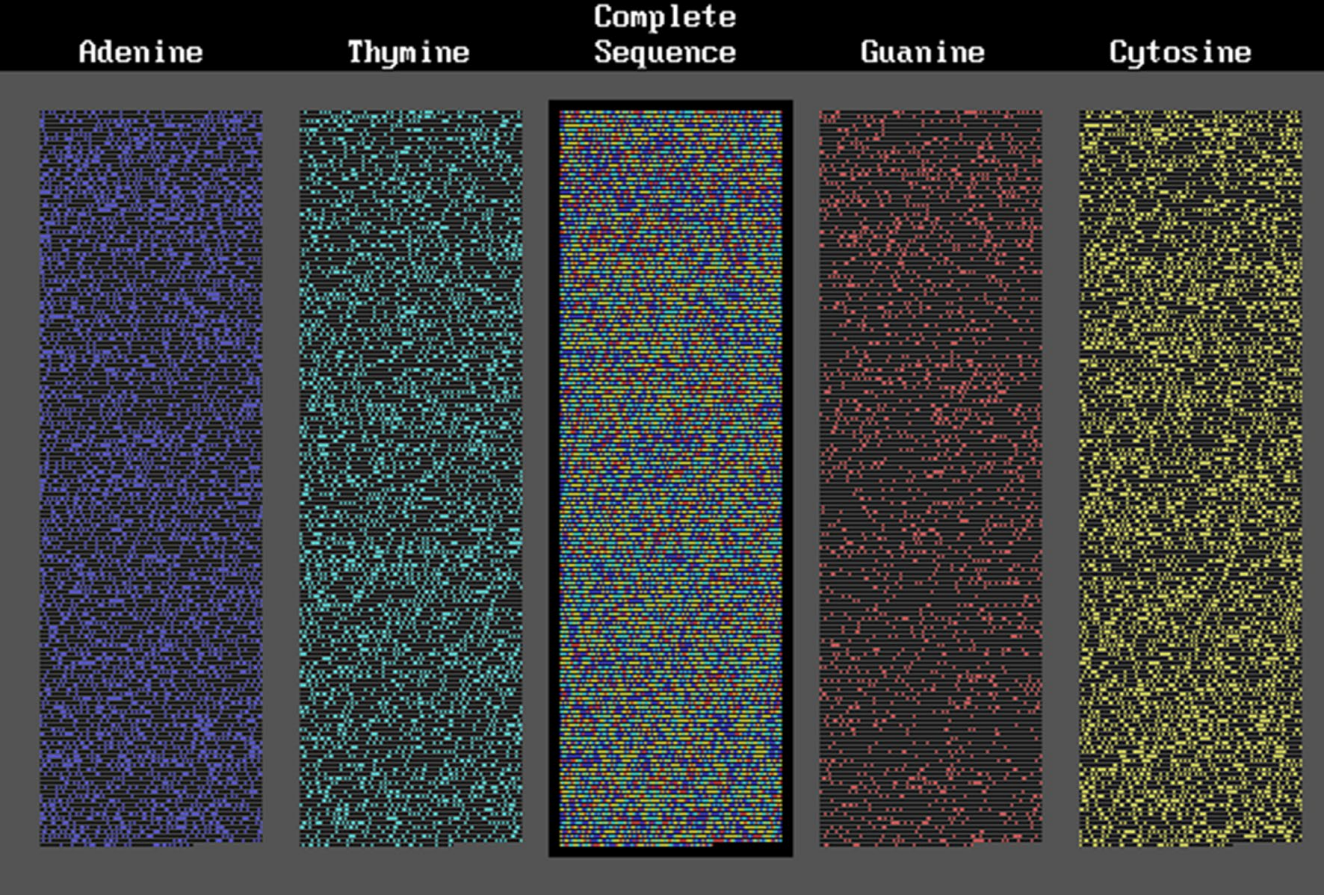
Human mtDNA

Table 3 shows the statistical analysis for four eukaryote DNA segments including the human mtDNA [GenBank accession number (GB-AN) = DQ523630; 16,569 bp] chosen to be compared with the already presented *D melanogaster* mtDNA. The number of sites between bases (separation) ranges from 0 to 26. A collagen gene was chosen because it codes for the periodic amino acid collagen molecule. This is the collagen type I alpha 2 gene (GB-AN = NM_000089, gene = COL1A2; 5411 bp); it was chosen because of its known periodicity due to the repetition of the amino acid triplet G-X-Y, where G is glycine and X and Y are other amino acids (often proline as X and 4-hydroxy-proline as Y); thus it has a periodicity of 9 nucleotides, or 9 Kper, that has been maintained for 800 million years [20] and has resisted a great number of mutations, some of which are known in any clinical genetic service [21]. This periodicity is produced because the codons for glycine are GGU, GGC, GGA and GGG; we use here the DNA that is homologous to the RNA, thus, the triplets are GGT, GGC, GGA and GGG, respectively. Proline and hydroxyproline (a post-translated hydroxylated proline) are coded by the same set of codons whose “coding” DNA is CCA, CCG, CCT and CCC respectively. The codons are not distributed equally in both glycine and proline; those ending in T produce a T-3 Kper. This periodic DNA segment was chosen also to test our programs; if they work they should show the largest $\chi_{9}^{2}$ value for 9 Kper (1° GG, 2°, 3°,… 9° GG) and a second for 3 Kper (T..T..T), as is described in Table 3 where the Chi squared values are rounded to integers. Collagen shows the expected 9 Kper of GG pairs with $\chi_{9}^{2}$ values near 1000 (P < 10^−116^) and 3 Kper of T-T pairs with values near 325 (P < 10^−37^); all the separations associated with significant deviations. Figure 2 shows this collagen DNA segment with the same nomenclature as for the HIV-1 figure. Periodicities are seen in a row or in several rows as a “kind of” rain falling from top-right to bottom-left or vice versa. A DNA segment from a worm (*C. elegans*; GB-AN = AY551966, gene = TRR-1; 12,503 bp) and from a fungus (*U. maydis*; GB-AN = AY124376, gene = BRH2; 7590 bp) of maize were added. Figure 3 shows the base distribution for the human mtDNA; this mtDNA presents the 3K periodicity like the *D. melanogaster* mtDNA ([8, 10]; Table 3), even though these genomes do not show the evident sequential periodicity seen in the collagen gene. The statistical analyses and the figures demonstrate that two different kinds of periodicity may produce the same result.

**Table 3:** Total $\chi_{9}^{2}$ value (in integers) of difference from randomness of dinucleotides from eukaryotes

	Human collagen			Human mtDNA			*C. elegans* TRR-1			*U. maydis* BRH2		
K	$\chi_{9}^{2}$	(MD)S	$\chi_{1}^{2}$	$\chi_{9}^{2}$	(MD)S	$\chi_{1}^{2}$	$\chi_{9}^{2}$	(MD)S	$\chi_{1}^{2}$	$\chi_{9}^{2}$	(MD)S	$\chi_{1}^{2}$
0	634	CG−	200	272	CG−	89	714	TA−	256	265	TA−	92
1	129	AA+	33	54	TC−	10	137	TC+	32	70	CA+	16
2	355	TT+	111	146	GG+	39	25	GG+	8	21	GG+	5
3	93	GG−	23	52	TG−	17	34	TT−	6	6	AA+	2
4	110	GG−	28	41	GT−	9	23	CT−	4	20	GT+	6
5	325	TT+	84	149	GG+	35	18	GA−	4	15	AC−	4
6	70	TG+	19	47	TG−	10	17	CG−	5	29	GC−	7
7	203	GC−	46	48	GT−	13	42	AT−	11	8	AG+	2
8	1058	GG+	457	91	GG+	21	44	TA−	7	17	AA+	5
9	198	CG−	46	62	GT+	14	20	CT+	6	15	AA+	3
10	84	GG−	22	23	GT−	5	20	TG−	4	19	GG−	4
11	341	TT+	70	153	TT+	31	86	TT+	28	21	GG+	8
12	114	GG−	36	51	GT+	18	15	CT+	4	6	CC−	1
13	86	GG−	26	41	GT−	7	8	GT+	2	11	AT−	4
14	337	TT+	96	80	TT+	17	31	GT−	9	17	GC−	3
15	66	TG+	18	43	GT+	8	18	CG−	4	12	GC−	4
16	156	GC−	42	31	GT−	6	14	CA+	5	9	TG+	2
17	975	GG+	425	86	GG+	23	13	TT+	4	13	TT+	4
18	159	CG−	47	53	GA−	11	14	TG+	6	12	AA+	2
19	84	TT−	18	27	GT−	8	37	GT+	15	8	CT+	1
20	325	TT+	100	106	GG+	27	36	TT+	10	4	AT−	1
21	97	GG−	29	46	TT−	11	32	GA+	4	18	GG+	4
22	80	GG−	23	46	AT+	10	42	AT−	15	7	AT+	1
23	316	TT+	85	64	TT+	17	30	GG+	8	13	GG+	3
24	72	GG−	20	32	GT+	10	9	TG+	2	16	TT+	5
25	194	GC−	53	30	GT−	10	21	TG−	3	5	CG+	1
26	1010	GG+	440	139	TT+	39	18	GG+	5	19	GG+	7

Bases separated by K sites, and $\chi_{1}^{2}$ contribution for the most different (from neutrality) pair (MD). Signs (S) indicate more (+) and less (−) observed than expected pairs

Human mtDNA shows high significant differences from randomness and a clear 3 Kper where largest values fluctuate near 100 (P < 10^−11^) and the others around 40 (P < 10^−7^, from tables). It is important to remark that this periodicity is different from that of the collagen gene. This is seen in the human mtDNA base distribution presented in Fig. 3, where no systematic periodicity is apparent (within a row or among rows as the mentioned “rain”), as it is in the collagen gene, even though a large significant 3 Kper was found by the test. Few “rain images” are seen; they may be compatible with the expected random distribution. The 3 Kper of the DNA segments is an intentionally searched coincidence.

The collagen gene has a sequence periodicity that includes two non-periodic extremes (see Fig. 2). If we divide the collagen gene into 8 equal sub-segments the first sub-segment does not present periodicities but a significant interaction; the 7th and the 8th sub-segments also did not present significant interactions or periodicities. From the 2nd to the 6th sub-segments significant interactions and significant 3 and 9 Kper were found. This 3K (that includes the 9 Kper) sequence periodicity produces an exactly equal and non-decaying set of $\chi_{9}^{2}$ values. The stochastic periodicity we found produces a fluctuating set of values that eventually may blur the periodicity and decays slowly but inexorably as K increases. It is evident that this collagen gene has interactive and periodic segments that are contiguous to non-interactive and non-periodic segments; this remarks the stochastic nature of these interactions and periodicities. The other two eukaryote DNA segments show less significant deviations from randomness; they did not present periodicity as clearly as the human mtDNA, although a smooth 3 Kper may be found. Figures for these last DNA segments are not presented.

#### Prokaryote DNA segments

**Fig. 4:**
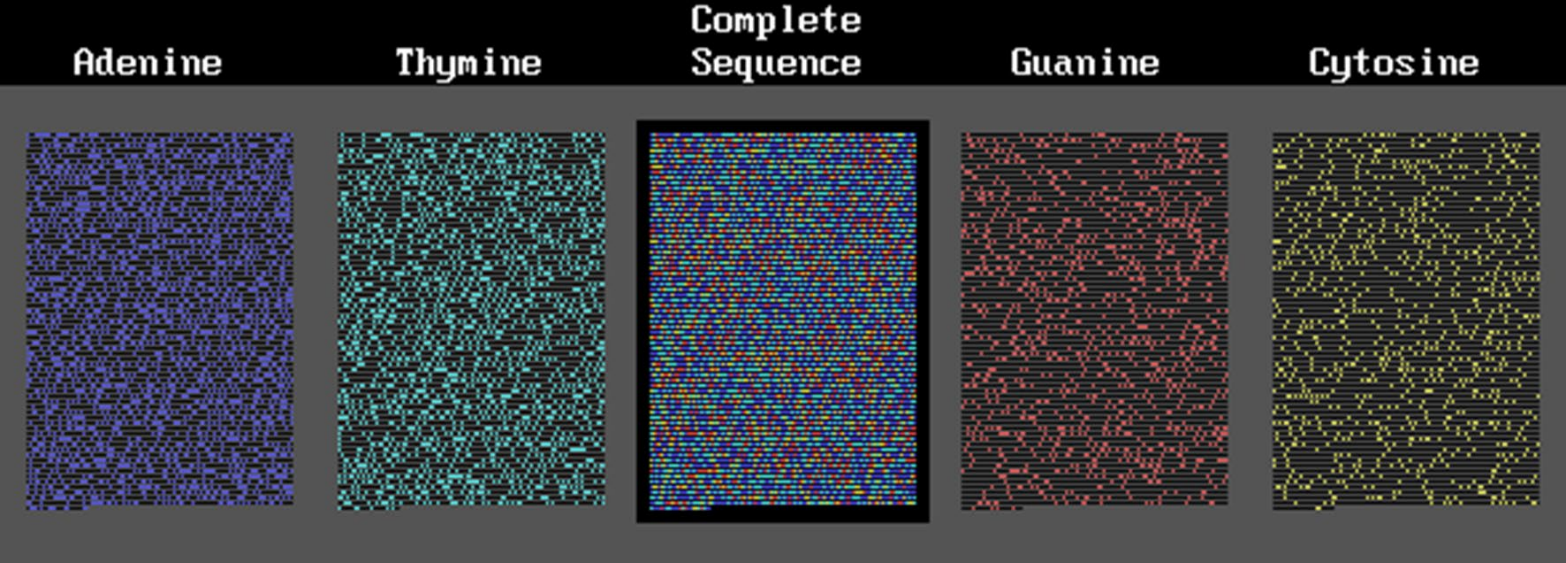
A gene from *Rickettsia prowazekii*

**Fig. 5:**
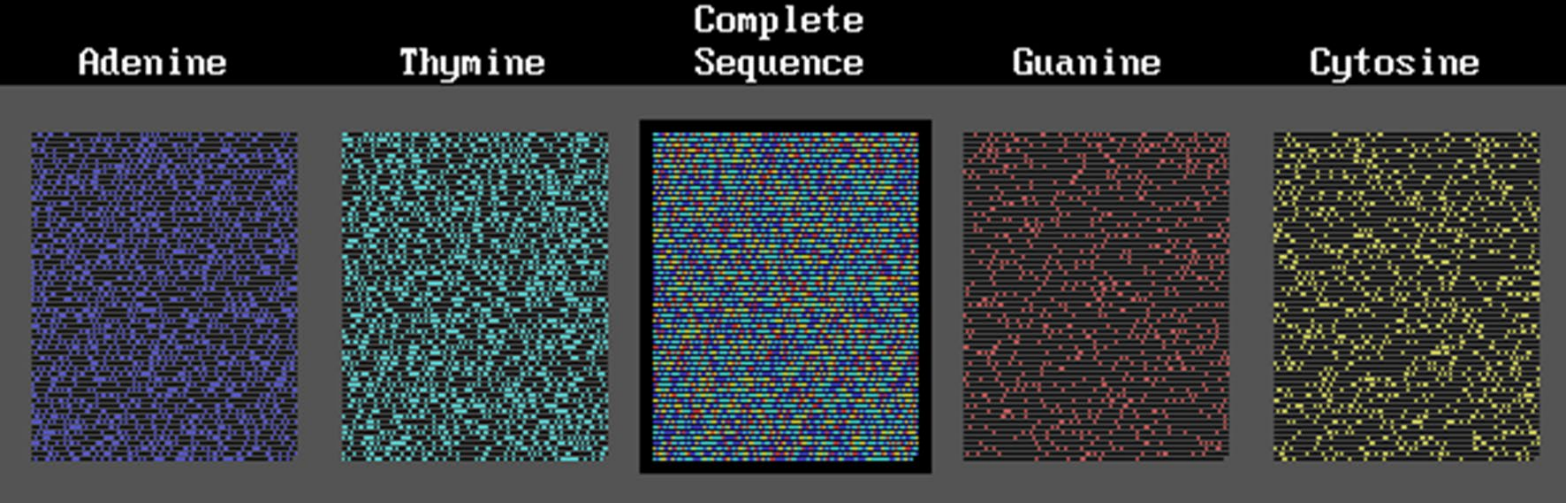
A gene from *Methanobrevibacter smithii*

Among prokaryotes, in bacteria a DNA region of *Deinococcus radiodurans* (GB-AN = AE000513, locus tag = DR 0687, REGION: 697364… 702340, 4977 bp) was chosen because of its resistance to radiation [22]. This bacterium repairs its genome once it has been cut in hundreds of segments after radiation. The origin of this resistance is not known, though several enzymes of DNA repair have been described [23, 24]. I hypothesized that a high internucleotide correlation along the whole genome could be a factor of this high capacity for fast genome repair. Also a gene from *Bacillus cereus* (GB-AN = NC_003909, gene rpoB, REGION: 108393… 111926, 3534 bp) and *Rickettsia prowazekii* (GB-AN = NC_000963, locus tag = RP451, REGION: 555011… 62033, 7023 bp) were chosen; the latter was examined because it is an intra-cytoplasmatic (in eukaryotes) organism and has a genome with traits of mtDNA [22, 25]. A DNA segment from the predominant archaea of the human gut, *Methanobrevibacter smithii* (GB-AN = CP000678, REGION: 249362–255559, 6198 bp corresponding to an adhesin-like protein, [22]) was chosen. Table 4 presents this analysis. We see the great selective (non-random) internucleotide interactions and the 3 Kper in the four DNA segments although some of them are blurred by the large non-periodic significant interactions. Figures 4 and 5 show the base distribution for *R. prowazekii* and *M. smithii*, respectively (those of *B. cereus* and *D. radiodurans* are not shown). Both figures have few and discrete “rains” mostly present in thymine of *R. prowazekii* and cytosine of *M. smithii*, but they did not present sequence periodicity in a row, thus sequence periodicities do not account for the huge significance of stochastic periodicities given in Table 4. Periodicities that are not so clear in Table 4 appear clearly in separations over 30 (not shown) and in the entire genomes (see Tables 6, 7). Thus we studied the interaction and periodicity in separations from K = 999 to K = 1008 (see Table 5). After 998 sites of separation, between the bases, the collagen conserved the 9 Kper and the 3 Kper, and the mtDNA its 3 Kper with lower significances; mtDNA seems to have had a shift in one site of separation. TRR-1 of *C. elegans* and BRH2 of *U. maydis* conserved their significance for deviation from randomness (more than one value over 17) but did not show periodicity. In the four prokaryotes both selective interactions and periodicity were clearly present.

**Table 4:** Total $\chi_{9}^{2}$ value (in integers) for differences from randomness of dinucleotides from prokaryote DNA segments

	*B. cereus* rpoB			*D. radiodurans* DR 0687			*R. prowazekii* RP451			*M. smithii* adhesin		
K	$\chi_{9}^{2}$	(MD)S	$\chi_{1}^{2}$	$\chi_{9}^{2}$	(MD)S	$\chi_{1}^{2}$	$\chi_{9}^{2}$	(MD)S	$\chi_{1}^{2}$	$\chi_{9}^{2}$	(MD)S	$\chi_{1}^{2}$
0	56	TA−	10	114	TG+	21	226	GG+	41	200	CG−	51
1	43	GC−	11	103	CC−	24	99	CG+	18	181	CG+	73
2	15	GG+	6	155	AT+	32	146	GT−	42	139	AC−	24
3	10	TG+	3	91	TC+	15	105	GC+	28	86	CA+	30
4	23	GG−	9	107	TG+	28	82	TC+	16	68	AC+	22
5	30	GG+	14	181	TT+	42	111	GG+	32	103	CC+	24
6	15	GG−	5	63	TA−	17	74	TC−	11	65	CA+	13
7	15	CC−	4	69	CT+	15	71	GT+	14	72	CG+	20
8	21	GG+	7	133	TA+	36	119	GT−	34	49	CC+	10
9	19	AA−	4	51	TT−	10	52	TG+	15	46	GC+	12
10	11	GT+	4	139	CT+	37	88	GT+	15	68	CG+	21
11	25	GG+	9	113	AA+	14	51	GG+	15	60	CC+	15
12	10	TG+	3	76	TA−	12	84	GC+	13	67	CA+	15
13	20	GT+	5	83	CA+	14	71	GT+	18	67	CG+	25
14	40	GG+	18	69	AT+	13	87	GT−	22	70	AA+	14
15	26	GG−	7	96	TC+	18	79	GC+	14	93	GC+	36
16	10	CT+	3	110	AG+	20	45	GT+	13	51	AA−	7
17	46	GG+	18	100	TT+	16	156	GG+	49	99	CC+	33
18	5	GG−	1	91	GT+	13	63	CT+	12	46	AA−	12
19	16	GT+	7	81	CT+	12	64	TC+	15	60	CG+	16
20	37	GG+	15	116	AC−	17	164	GT−	41	129	CC+	38
21	6	GG−	3	86	TA−	26	57	TG+	14	42	GC+	16
22	3	GG−	1	83	CT+	10	89	GT−	21	59	CG+	16
23	29	GG+	10	179	CA−	25	131	GG+	41	85	CA−	16
24	12	AG+	3	39	CG+	7	84	TG+	15	64	CA+	15
25	25	GG−	8	85	AT−	14	35	GT+	7	98	AC+	21
26	30	GG+	14	96	TT+	15	207	GT−	45	77	CA−	18

Bases separated by K sites, and $\chi_{1}^{2}$ contribution for the most different (from neutrality) pair (MD). Signs (S) indicate more (+) and less (−) observed than expected pairs

**Table 5:** $\chi_{9}^{2}$ value for difference from randomness and its $\chi_{1}^{2}$ for the most distant pair. Eukaryotes, prokaryote DNA segments and human mtDNA

Sep	$\chi_{9}^{2}$	(MD)S	$\chi_{1}^{2}$	$\chi_{9}^{2}$	(MD)S	$\chi_{1}^{2}$	$\chi_{9}^{2}$	(MD)S	$\chi_{1}^{2}$	$\chi_{9}^{2}$	(MD)S	$\chi_{1}^{2}$
Eukaryotes												
K+1	Human collagen			Human mtDNA			*C. elegans TRR*-*1*			*U. maydis* BRH2		
999	452	GG+	188	7	GT+	3	8	GA−	1	8	GA+	3
1000	64	CG−	22	15	AT+	3	33	TG+	11	4	CG−	1
1001	52	TT−	17	15	TT+	7	4	TT−	1	5	AC−	1
1002	153	TT+	48	8	TG−	4	21	GG+	6	11	TC+	4
1003	42	GG−	12	22	GC+	4	13	GA+	3	21	TC−	7
1004	55	GG−	16	12	AG−	4	29	TA+	8	9	CG+	3
1005	139	TT+	44	6	CT−	2	14	TT+	5	18	TG−	3
1006	44	TG+	8	30	TT−	11	14	TG+	3	5	TC−	2
1007	89	GC−	27	3	AT−	0	8	TA+	2	11	GA−	3
1008	467	GG+	196	16	GT+	6	21	TT+	8	9	AC+	3

Prokaryotes												
	*B. cereus*			*D. radiodurans*			*R. prowazekii*			*M. smithii*		
999	28	GG+	11	104	TT+	15	122	TG−	27	86	CC+	21
1000	7	CT−	2	41	TC+	8	52	GC+	12	55	CA+	17
1001	9	AG+	3	69	AT−	11	45	TC+	8	47	AC+	10
1002	27	GT−	9	122	AT+	16	119	TT+	30	96	CC+	36
1003	13	TC+	4	58	GT+	10	59	TG+	12	59	CA+	16
1004	12	TG+	2	53	AT−	14	69	GT+	13	28	AC+	8
1005	43	GG+	8	122	TT+	27	129	TG−	29	90	CA−	28
1006	13	CG+	2	65	TA−	12	77	GG−	15	58	GC+	10
1007	10	GT+	2	72	CT+	14	40	CG+	8	62	AC+	15
1008	29	GG+	10	94	TT+	18	90	GT−	21	95	AA+	19

*S* signs indicate more (+) and less (−) observed than expected pairs. Chi square values rounded to integers. *Sep* separation of K + 1 sites. MD pair most different from neutrality

Among viruses, HIV-1 was already studied [2, 7, 8, 10]; a large internucleotide interaction was found but not a clear periodicity.

### Larger genomes and separations between bases

**Fig. 6:**
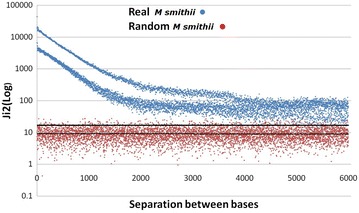
Comparison of $\chi_{9}^{2}$ values between the random and the real *M. smithii* genomes

**Fig. 7:**
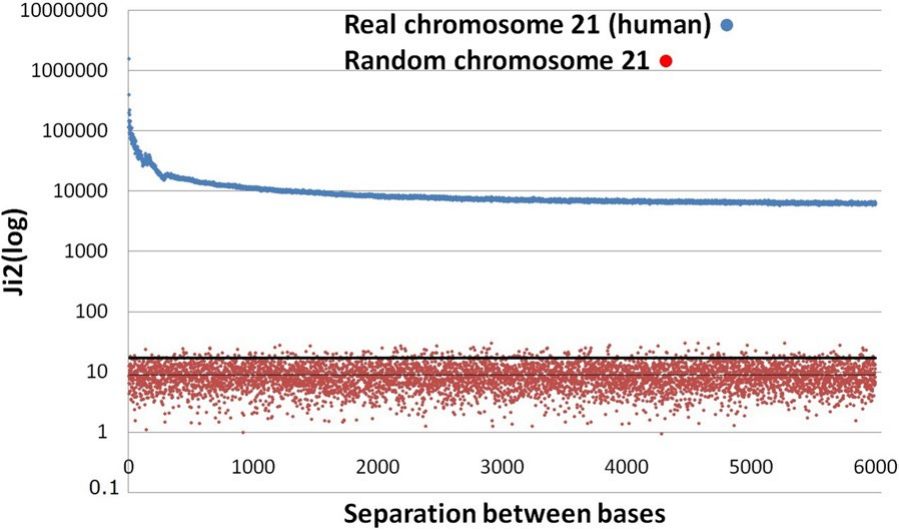
Comparison of $\chi_{9}^{2}$ values between the random and the real human chromosome 21

The analysis was extended to the complete genome of *Methanobrevibacter smithii* (archaea; GenBank, *M. smithii*: NC_009515.1; 1,853,160 bp) and human chromosome 21 (HCh21, GB-AN = NC_000021.9; 46,709,983 bp from which only 40,088,619 could be included as A, T, G or C). This is shown in Table 6 with separations between 0 and 26 sites. As noted, we had not found clear periodicities in virus and eukaryote DNA segments [2, 8] but HCh21 showed 2K and 6K periodicities ([12], this article). The 3K periodicity is evident in the *M. smithii* genome. The minimum total significant values of the base to base interaction was $\chi_{9}^{2} = 2683.6$ (at Sep 8) in the case of *M. smithii* (*P* < 10^−315^) and 63,342.0 (at Sep 33) for HCh21 (*P* < 10^−7463.9^). The maximal significance implies a probability less than 10^−180,000^ a value that leads us to think of a meta-intelligent design (Laplacian vast intelligence?) where everything, in the universe, is determined since the beginning. We have reviewed around 30 prokaryote genomes and 30 mtDNA that presented a high internucleotide interaction and 3K periodicity. In about 10 DNA segments of eukaryote genomes we found large interactions but we did not find clear periodicities except in the collagen genes as was mentioned. However, we found a 3 Kper in the six chromosomes of *C. elegans* and some periodicities in other human chromosomes. Figures 6 and 7 show $\chi_{9}^{2}$ values for *M. smithii* and HCh21, respectively, until Sep 6000. These figures show in red the $\chi_{9}^{2}$ values for randomly-constructed *M. smithii* and HCh21 DNAs for a visual statistical comparison. These figures were constructed during a research unit of under graduated students [12]; the human chromosome 21 was an old version of its q-arm and less than 33,000,000 bp, and the *M. smithii* was also an old version in which the genome had a small but important proportion of non identified bases; HCh21 showed significant interactions with separations over 15 millions nucleotide sites. The results of both versions of *M. smithii* are completely comparable; some differences were found in the two versions of HCh21 (this is due to the number of bp analyzed and the proportion of unknown bases), in relation to data presented in Table 6, but not at a level to change the results presented in Fig. 7. These figures are presented to acknowledge the hard and devoted work of these students. Table 6 was constructed with the updated information. It is important to know that these randomly constructed genomes yielded $\chi_{9}^{2}$ values completely in agreement with the expected theoretical values; this gives strong confirming evidence to our method.

**Table 6:** Internucleotide interactions measured by a $\chi_{9}^{2}$ test in dinucleotides

Sep	*M. smithii*			Human Ch21		
K+1	$\chi_{9}^{2}$	1° DN	$\chi_{1}^{2}$	$\chi_{9}^{2}$	1° DN	$\chi_{1}^{2}$
1	45,469.3	CG(−)	13,210.2	1,885,266.8	CG(−)	882,286.7
2	17,658.5	CG(+)	7962.4	*524,989.7*	AA(+)	*108,841.0*
3	*17,489.1*	*CC*(+)	*5905.4*	143,071.9	GG(+)	30.632.7
4	5333.5	CG(−)	1125.0	*223,073.3*	AA(+)	*36,261.4*
5	4734.2	GC(−)	1125.0	130,240.9	CC(+)	21,195.2
6	*13,978.8*	*CC*(+)	*5220.7*	*232,327.6*	CC(+)	*46,338.4*
7	4204.9	GC(+)	1147.2	137,408.0	GG(+)	36,654.3
8	2683.6	CG(+)	988.2	*200,806.8*	GG(+)	*35,345.5*
9	*16,230.4*	*CC*(+)	*4805.5*	168,931.2	GG(+)	38,267.5
10	3621.9	GC(+)	1247.9	*179,941.9*	CC(+)	*30,987.9*
11	3181.3	CG(+)	1100.9	97,021.9	AA(+)	12,523.8
12	*18,816.5*	*CC*(+)	*5921.3*	*172,146.7*	TT(+)	*28,086.2*
13	2913.5	GC(+)	1104.4	118,939.4	TT(+)	21,745.2
14	3830.5	CG(+)	1487.8	*140,757.2*	AA(+)	*25,256.0*
15	*14,486.0*	*CC*(+)	*4624.5*	108,724.8	CC(+)	19,839.1
16	3208.5	GC(+)	1196.6	*125,307.4*	AA(+)	*17,579.7*
17	3015.7	CG(+)	1130.7	73,618.5	CC(+)	10,267.1
18	*15,874.2*	*CC*(+)	*5181.5*	*100,806.2*	*CC*(+)	*20,558.8*
19	3370.1	GC(+)	1349.0	76,660.2	GG(+)	12,402.2
20	2817.1	CG(+)	1137.0	*102,585.4*	*AA*(+)	*14,239.5*
21	*17,678.9*	*CC*(+)	*5389.7*	81,732.7	AA(+)	10,331.6
22	3581.5	GC(+)	1454.3	*86,665.1*	*GG*(+)	*14,559.0*
23	3186.5	CG(+)	1095.2	84,086.3	AA(+)	13,354.2
24	*14,742.2*	*CC*(+)	*4673.1*	*138,427.5*	*CC*(+)	*23,014.6*
25	3006.4	GC(+)	938.3	111,182.0	TT(+)	22,494.8
26	3251.2	CG(+)	1170.9	89,507.1	TT(+)	15,788.0
27	*14,054.4*	*CC*(+)	*4473.3*	101,184.2	GG(+)	21,244.9

*M. smithii* genome and human chromosome 21

*Sep* number of sites (plus one) between the first and second base of a dinucleotide, *1*° *DN* the most significant (deviation from neutrality) pair among the 16. (+) or (−) more or less observed than expected pairs, respectively. Italics indicates periods

#### Complete prokaryote genomes

We have analyzed more than 40 prokaryote genomes; four are presented here. The complete genomes of *B. cereus* (GenBank, *B. cereus* NC7401: AP007209.1; 5,221,581 bp), *D. radiodurans* (GenBank *D. radiodurans* R1 chromosome 1: NC_001263.1; 2,648,638 bp), *R. prowazekii* (Gen Bank, *R. prowazekii*: NC_000963; 1,111,523 bp), and *M. smithii* (see above), whose analyses were presented in Table 6 and Fig. 6. We see in the three genomes of Table 7 the high internucleotide interaction and 3K periodicity, again, with enormous differences to neutrality. The most significant total deviation from neutrality was found in *D. radiodurans* (chromosome 1), even though it is not the largest genome. The total 3K periodicity of *M. smithii*, *B. cereus* and *R. prowazekii* is constructed with the highest significant head of CC(+) pairs followed by two less significant tails of GC(−) or CG(−) pairs. In *M. smithii* the head and the tails have positive deviations from neutrality; in *B. cereus* the head CC is always positive and the tails are always negative; in *R. prowazekii* CC are positive and tails are positive or negative. In *D. radiodurans* the head is TT(+) or AA(+) pairs followed by tails of AT(−) or TA(−) pairs, showing a very different phylogenetic origin than the other bacteria. These similarities and differences could be taken as a new criterion to construct phylogenies; however we still do not have a method to evaluate the evolutionary meaning of the differences. For example, the pair CC(+) is very close to its complementary GG(+) pair but they are evolutionarily very different from the CC(−) or GG(−) pairs. Sometimes we do not find a clear periodicity with one pair as the head and the two tails, but if we examine the complementary pairs, a clear periodicity is found in this significance or in the following ones.

**Table 7:** Total $\chi_{9}^{2}$ value for differences from randomness of dinucleotides of prokaryote genomes

Sep	*B. cereus*			*D. radiodurans*			*R. prowazekii*		
K+1	$\chi_{9}^{2}$	(MD)S	$\chi_{1}^{2}$	$\chi_{9}^{2}$	(MD)S	$\chi_{1}^{2}$	$\chi_{9}^{2}$	(MD)S	$\chi_{1}^{2}$
1	52,775.3	TA−	13,986.2	53,841.9	TA−	18,564.7	12,165.2	GC+	6452.1
2	41,043.3	GC−	10,401.0	6789.9	AT−	1175.8	2547.1	GC−	894.9
3	42,065.9	CC+	7601.9	38,411.4	TT+	4899.4	6282.7	CC+	1217.4
4	218,522	CG−	6086.2	18,543.1	TA−	4258.9	1845.1	CG−	444.3
5	7566.5	GC−	2818.3	17,156.6	CG−	4336.5	1951.9	GC−	955.8
6	20,901.5	CC+	6350.3	38,358.8	TT+	5161.0	3433.9	CC+	1088.9
7	8446.0	CG−	3334.7	12,489.1	TA−	3005.9	1292.4	CG−	522.5
8	3225.5	GC−	1.381.1	11,437.2	AT−	2993.4	1044.0	GC−	398.4
9	21,854.9	CC+	6071.2	37,282.8	AA+	7180.4	4799.8	CC+	1020.7
10	6589.2	CG−	2578.7	10,136.9	TA−	3065.7	945.1	CG−	355.0
11	5279.3	GC−	2167.0	9152.0	AT−	2722.3	1053.5	CG+	317.4
12	26,475.6	CC+	7117.3	47,310.8	TT+	8069.6	4791.1	CC+	1220.7
13	3076.9	CG−	903.7	10,516.9	TA−	2962.6	993.8	GC+	320.1
14	5795.4	GC−	1923.2	11,167.0	AT−	2546.7	1316.5	CG+	380.2
15	20,371.8	CC+	6091.0	36,880.5	TT+	5222.8	3788.8	CC+	1120.5
16	3547.9	CG−	1373.9	12,422.9	TA−	2664.7	908.9	CG−	315.9
17	4049.2	GC−	1814.6	11,416.3	AT−	2668.5	825.0	GC−	259.8
18	21,761.5	CC+	6791.3	36,418.3	TT+	4659.1	3899.4	CC+	1054.4
19	5049.3	CG−	2065.2	11,337.4	TA−	2340.1	1102.6	GC+	365.6
20	3987.2	GC−	1363.8	9893.4	AT−	2764.1	742.1	CG+	235.9
21	24,493.5	CC+	6523.1	41,508.1	TT+	7240.1	4701.4	CC+	1155.7
22	5145.9	CG−	1714.5	10,525.4	TA−	2665.5	1169.1	GC+	387.7
23	3732.6	GC−	1130.0	10,332.5	AT−	2729.9	861.4	CG+	276.3
24	21,564.0	CC+	6302.9	37,752.4	TT+	6432.9	4213.2	CC+	1078.3
25	3810.5	CG−	1343.6	11,857.5	TA−	2773.7	995.3	CG−	334.7
26	3608.2	GC−	1447.3	11,438.2	AT−	2854.9	930.1	CG+	270.3
27	20,132.5	CC+	6016.4	34,145.0	TT+	5348.5	3833.9	CC+	971.9

Bases separated (Sep) by K sites, and $\chi_{1}^{2}$ contribution for the most different (from neutrality) pair (MD). Signs (S) indicate more (+) and less (−) observed than expected pairs

The structure of the periodicity in the mtDNA of *D melanogaster* has been elucidated for each pair [13]. All pairs presented this 3K periodicity that is more significant in pairs where G or C is involved. The periodicity fits the complementariness of bases that is similar in GG and CC; GC and CG; AT and TA and so on. This was expected because evolution simultaneously involves the pair in one strand and the complementary pair in the other strand. Selection operates in the tetrad C–G//G–C where the 3′–5′ sense in the strands runs in the opposite direction.

### Phylogenetic analyses

We have applied the panel of this periodicity and significance of dinucleotides to phylogenetic analysis and the power of discrimination of taxa is enormous; we may imagine a table of 2000 sites of separation and 16 significance values that gives the phylogenetic relationships directly. The study of some bacteria of the *Phylum Firmicutes* may illustrate this method. This phylum presents three classes*: Clostridia*, *Mollicutes* and *Bacilli* [22]. Following the phylogeny published for *Bacilli* by Tremblay-Savard et al. [26] we examined the genomes of 20 *Bacilli* belonging to strains of *B. cereus*, *B. anthracis* and *B. thuringiensis*, 4 strains of *B. subtilis*, and strains of *B. weihenstephanensis*, *B. cytotoxicus* and *B. atrophaeus*, and *B. selenetireducens*; we added *Staphylococcus aureus*, *Lactobacillus casei*, *Clostridium botulinum* and *Mycoplasma hominis* (*Mollicutes*) which are non-*Bacillus Firmicutes*. Table 8 presents the periodicities found in the most deviated (from randomness) pair (first significance), in separations from 0 to 18. The 20 *Bacilli* species (strains) showed an identical pattern of periodicities beginning with TA(−), GC(−) and CC(+), and then with triplets of CC(+), CG(−) and GC(−) with the only exception of *B. weihenstephanensis* at separation 12 with GC(+) as the most deviated pair instead of CG(−); however, at the second significance this bacterium presented the CG(−) pair, while the other bacilli present the GC(+) pair. The other chosen *Firmicutes* including *B. subtilis* are largely different from these bacilli, even though *S. aureus* and *C. botulinum* seem to have converged to a similar pattern of periodicities. The apparent convergence of *M. hominis* (*Mollicute*) is only in the name of dinucleotides, because it has GC(+) and CG(+) pairs instead of their negative counterparts. The homogeneity of *B. anthracis*, *B. thuringiensis* and *B. cereus* leaves space for some heterogeneity as we advance to lower significances or increase the number of separations. This method allows study selective processes such as convergence and intra nuclear chromosome comparative evolution. The method appears complementary to classical sequence methods, but it is completely founded on the mutation (forward and backward)—selection equilibrium and not on neutral or nearly-neutral models [3, 4].

**Table 8:** Periodicities found in the first significant pair in the 19 initial separations among bacteria of the Firmicutes group

S	*B. thur*	*B. cer*	*B. ant*	*B. wei*	*B. cyto*	*B. subt*	*B. atro*	*B. sele*	*Sta. au*	*La. ca*	*Clo. bo*	*My. ho*
0	TA−	TA−	TA−	TA−	TA−	TA−	TA−	TA−	TA−	TA−	CG−	TA−
1	GC−	GC−	GC−	GC−	GC−	CG+	CG+	CG+	GT+	CC−	TA−	CC−
2	CC+	CC+	CC+	CC+	CC+	AT−	AT−	AT−	GG+	GC−	CC+	GG+
3	CG−	CG−	CG−	CG−	CG−	AT−	AT−	TA−	CG−	TA−	CG−	GC+
4	GC−	GC−	GC−	GC−	GC−	AT−	AT−	AT−	GC−	AC+	GC−	CG+
5	CC+	CC+	CC+	CC+	CC+	GG+	GG+	GG+	CC+	CC+	CC+	CC+
6	CG−	CG−	CG−	CG−	CG−	CG−	CG−	AC+	CG−	AA−	CG−	GC+
7	GC−	GC−	GC−	GC−	GC−	AT−	AT−	AT−	GC−	AT−	GC−	CG+
8	CC+	CC+	CC+	CC+	CC+	GG+	GG+	GG+	CC+	CC+	CC+	GG+
9	CG−	CG−	CG−	CG−	CG−	TA−	TA−	TA−	CG−	CA+	CG−	GC+
10	GC−	GC−	GC−	GC−	GC−	AT−	AT−	AT−	GC−	AC+	GC−	CG+
11	CC+	CC+	CC+	CC+	CC+	GG+	GG+	GG+	GG+	CC+	CC+	GG+
12	CG−	CG−	CG−	GC+	CG−	TA−	CG+	CG+	GC+	AT+	CG−	GC+
13	GC−	GC−	GC−	GC−	GC−	CG+	CG+	TA+	CG+	TA+	GC−	CG+
14	GG+	CC+	CC+	CC+	CC+	GG+	GG+	GG+	GG+	CC+	CC+	GG+
15	CG−	CG−	CG−	CG−	CG−	CG+	CG+	GA+	CG−	AT+	CG−	GC+
16	GC−	GC−	GC−	GC−	GC−	GC+	GC+	AT−	GC−	TA+	GC−	CG+
17	CC+	CC+	CC+	CC+	CC+	GG+	GG+	GG+	CC+	CC+	CC+	GG+
18	CG−	CG−	CG−	CG−	CG−	TA−	TA−	AT+	CG−	AT+	CG−	GC+

Bacterial strains of the Firmicutes group. *Bacillus: thuringiensis, cereus, anthracis, weihenstephanensis, cytotoxicus, subtilis, atrophaeus, selenitireducens. Staphylococcus aureus; Lactobacillus casei; Clostridium botulinum; Mycoplasma hominis*. Separation (S) from 0 to 18 nucleotide sites

However, we need to study in depth the evolutionary meaning of differences and similarities in this panel of separations-significances before applying these periodicities to consistent phylogenic analyses. The examination of known phylogenic groups could show us the nature of these differences and similarities. This is the aim of future studies with known taxa.

## Conclusions

The nucleotide bases in the DNA molecule are distributed enormously different from a random or neutral distribution either in longitudinal segments or in dinucleotides. This selective distribution has been maintained over millions of cell generations making the neutral or nearly-neutral models of evolution untenable or simply impossible. Only the synthetic theory of evolution can account for these facts. The study of the distribution of bases of dinucleotides separated by 0 (contiguous), 1, 2… K shows a significant and huge selective internucleotide interaction and a periodicity of the statistical value of the deviation from randomness. This interaction and periodicity is observed in genomes with K greater than 1000 and in human chromosome 21 with K over 10 millions. The most important conclusions are that a base co-evolves with all the other bases of the genome or there is a genome co-adaptation or co-selection of the bases of this genome. This periodicity and the different significance of the 16 dinucleotides may be used to construct phylogenies with a completely different approach than phylogenies made using sequence differences.

## Abbreviation

HCh21: *Homo sapiens* chromosome 21
